# Identifying Key Genes and Functionally Enriched Pathways in Sjögren’s Syndrome by Weighted Gene Co-Expression Network Analysis

**DOI:** 10.3389/fgene.2019.01142

**Published:** 2019-11-13

**Authors:** Qiuming Yao, Zhenyu Song, Bin Wang, Qiu Qin, Jin-an Zhang

**Affiliations:** ^1 ^Department of Endocrinology, Jinshan Hospital of Fudan University, Shanghai, China; ^2 ^Department of Urology, Jinshan Hospital of Fudan University, Shanghai, China; ^3 ^Department of Endocrinology, Shanghai University of Medicine & Health Sciences Affiliated Zhoupu Hospital, Shanghai, China

**Keywords:** Sjögren’s syndrome, weighted gene co-expression network analysis (WGCNA), hub gene, biological process, gene set enrichment analysis

## Abstract

**Purpose:** Sjögren’s syndrome (SS) is an autoimmune disease characterized by dry mouth and eyes. To date, the exact molecular mechanisms of its etiology are still largely unknown. The aim of this study was to identify SS related key genes and functionally enriched pathways using the weighted gene co-expression network analysis (WGCNA).

**Materials and Methods:** We downloaded the microarray data of 190 SS patients and 32 controls from Gene Expression Omnibus (GEO). Gene network was constructed and genes were classified into different modules using WGCNA. In addition, for the hub genes in the most related module to SS, gene ontology analysis was applied. The expression profile and diagnostic capacity (ROC curve) of interested hub genes were verified using a dataset from the GEO. Moreover, gene set enrichment analysis (GSEA) was also performed.

**Results:** A total of 1483 differentially expressed genes were filtered. Weighted gene coexpression network was constructed and genes were classified into 17 modules. Among them, the turquoise module was most closely associated with SS, which contained 278 genes. These genes were significantly enriched in 10 Gene Ontology terms, such as response to virus, immune response, defense response, response to cytokine stimulus, and the inflammatory response. A total of 19 hub genes (GBP1, PARP9, EPSTI1, LOC400759, STAT1, STAT2, IFIH1, EIF2AK2, TDRD7, IFI44, PARP12, FLJ20035, PARP14, ISGF3G, XAF1, RSAD2,LY6E, IFI44L, and DDX58) were identified. The expression levels of the five interested genes including EIF2AK2, GBP1, PARP12, PARP14, and TDRD7 were also confirmed. ROC curve analysis determined that the above five genes’ expression can distinguish SS from controls (the area under the curve is all greater than 0.7). GSEA suggests that the SS samples with highly expressed EIF2AK2 or TDRD7 genes are correlated with inflammatory response, interferon α response, and interferon γ response.

**Conclusion:** The present study applied WGCNA to generate a holistic view of SS and provide a basis for the identification of potential pathways and hub genes that may be involved in the development of SS.

## Introduction

Sjögren’s syndrome (SS), a systemic autoimmune disease, is characterized by lymphocyte infiltration in exocrine glands, which further leads to destruction of their function (Fox, 2005). The SS occurs ten times more frequently in females than in males and it is most common in women aged between 40 and 60 years (Qin et al., 2015). SS is clinically characterized by keratoconjunctivitis sicca (dry eyes) and xerostomia (dry mouth) and may be accompanied by multi-organ systemic manifestations (Fox, 9482). Xerostomia can hinder eating, speaking, and swallowing, and cause rampant caries, all of which largely compromise quality of life for SS patients. SS that occurs without any other autoimmune diseases was defined as primary Sjögren’s syndrome (pSS) (Molano-Gonzalez et al., 2018). Furthermore, SS can occur in association with other autoimmune diseases, such as autoimmune thyroid diseases (AITD), systemic lupus erythematosus (SLE), and rheumatoid arthritis (RA) (Salliot et al., 2007; Anaya et al., 2016; Alani et al., 2018). Literatures have reported that several microRNAs were abnormally expressed in SS, indicating that they may be involved in the pathogenesis of SS and some microRNAs can also be used as diagnostic biomarkers of SS such as miR-146a, miR-768-3p, and miR-574 (Chen et al., 2015; Cha et al., 2018). A recent study also found that increased ligand for glucocorticoid-induced TNFR family-related protein (GITRL) plays a critical role in attenuating the function of myeloid-derived suppressor cells and can exacerbate SS (Tian et al., 2019). However, to our best knowledge, the etiology and progression of SS are still unclear to date.

Weighted gene co-expression network analysis (WGCNA) can be used to explore the gene-network signature associated with complicated diseases (Langfelder and Horvath, 2008). WGCNA can integrate gene expression and trait data effectively to identify functional pathways and candidate biomarkers (Presson et al., 2008). WGCNA has been reported to be applied to investigate the gene-network signature, co-expression modules, and hub genes involved in some autoimmune diseases, such as type 1 diabetes (Riquelme Medina and Lubovac-Pilav, 2016), rheumatoid arthritis (Ma et al., 2017), inflammatory bowel disease (Li et al., 2016), and Graves’ disease (Shao et al., 2018). There is possibility that WGCNA can also be useful to identify the gene-network signature and hub genes associated with SS and get deep understanding of its pathogenesis. Hub gene is a gene that interacts with many other genes in gene networks and usually plays a key role in biological processes and gene regulation (Yu et al., 2017). Up to now, our study is the first to use WGCNA analysis to explore the gene-network signature of peripheral blood related to SS with sample size more than 200. Furthermore, for the hub genes in the most related module to SS, gene ontology analysis was also applied to explore their potential functions. What’s more, gene set enrichment analysis for single gene was performed for selected hub genes in order to find the associated gene sets.

## Materials and Methods

### Data Collection and Preprocessing

We downloaded mRNA expression profiles of human SS from the Gene Expression Omnibus (GEO) database. In our study, GSE51092 was used to construct co-expression networks and identify hub genes related to SS. The microarray dataset provided gene expression profile in whole peripheral blood from 190 SS patients and 32 controls (Lessard et al., 2013). According to the data processing information of GSE51092, each dataset was normalized independently using Robust Multiarray Average (RMA) followed by log2 transformation and quantile normalization. ComBat was subsequently applied to the combined dataset to adjust for batch effect.

### Differentially Expressed Genes Screening

We screened the differentially expressed genes (DEGs) between SS patients and controls in the expressing data using the “limma” R package. The significance analysis of microarrays (SAM) was utilized to select significantly changed genes with false discovery rate (FDR) <0.05 and log2 fold change (FC) ≥1.

### Construction of Co-Expression Network

The co-expression network of the DEGs was constructed based on GSE51092 microarray dataset by the R package “WGCNA” (Langfelder and Horvath, 2008). The soft-thresholding power we chose was eight when 0.8 was used as the correlation coefficient threshold, and 10 was chosen as the minimum number of genes in modules. To merge possible similar modules, we defined 0.2 as the threshold for cut height.

### Functional Enrichment Analysis

To obtain further insights into the function of the DEGs in the module most related to SS, we referred to the Database for Annotation, Visualization and Integrated Discovery (DAVID) (https://david.ncifcrf.gov/home.jsp/) to perform the Gene Ontology analysis (Huang da et al., 2009; Huang da et al., 2009). P < 0.05 was set as the cut-off. The R package “GOplot” was adopted to show the results graphically.

### Hub Genes Identification

In the module-trait correlation analysis, hub genes were considered as genes with gene significance greater than 0.4 and high module group members (MM) (weighted correlation index > 0.9), indicating a significant correlation with some clinical features.

### Hub Genes Validation and Efficacy Evaluation

Among the hub genes, five genes (EIF2AK2, GBP1, PARP12, PARP14, TDRD7) of interest that have not been studied in SS were further validated in another two datasets GSE84844 (Tasaki et al., 2017) and GSE66795 (James et al., 2015) downloaded from GEO database. In the GSE84844 dataset, 30 SS patients and 30 healthy individuals were recruited and the RNA was extracted from their peripheral blood mononuclear cells (PBMCs). The data of GSE84844 were analyzed with the frozen robust multiarray analysis (fRMA) using Bioconductor with default settings and absence/presence calls were estimated by Microarray Suite version 5.0 (MAS 5.0). In the GSE66795 dataset, the total RNA was extracted from the peripheral whole blood of 131 SS patients and 30 healthy controls. Also, ROC curve was plotted and AUC was calculated with “pROC” package to evaluate the capability of selected genes to distinguish SS patients and controls.

### Gene Set Enrichment Analysis

To further explore the potential function of the selected hub genes in SS, gene set enrichment analysis (GSEA) for single hub gene was performed. In the dataset GSE51092, according to the median expression level of hub genes, 190 SS samples were divided into two groups. The R package “clusterprofiler” was utilized to conduct GSEA. The h.all.v6.2.sytmbols.gmt in Molecular Signatures Database (MSigDB) was selected as the reference gene set, and P adjusted value < 0.05 was chosen as the cut-off criteria.

### Statistical Analysis

The statistical significance of differences between the two groups was analyzed using non-parametric test or t test based on data distribution characteristics. All analyses were conducted using software R3.5.3. P value < 0.05 was considered statistically significant.

## Results

### Differentially Expressed Genes Between Sjögren’s Syndrome and Normal Controls

A total of 1,483 differentially expressed genes were identified and selected for subsequent analysis. The top 20 up-regulated genes and 10 down-regulated genes identified in the gene expression microarray study of 190 SS patients and 30 controls were shown in **Table 1**.

**Table 1:** The top 20 up-regulated genes and 10 down-regulated genes identified in the gene expression microarray study of 190 SS patients and 30 controls.

Gene	logFC	P.Value	adj.P.Val
EPSTI1	1.532429	7.80E-10	1.06E-06
LOC400759	1.255257	8.21E-10	1.06E-06
IFI44	1.724777	1.26E-08	7.25E-06
CXCL10	1.533126	1.42E-08	7.25E-06
FLJ20035	1.12229	1.60E-08	7.27E-06
XAF1	1.204912	4.07E-08	1.46E-05
RSAD2	2.177534	4.20E-08	1.46E-05
TTC21A	1.036592	4.28E-08	1.46E-05
LY6E	1.47236	5.69E-08	1.78E-05
IFI44L	2.175133	5.84E-08	1.78E-05
LAMP3	1.702514	9.52E-08	2.49E-05
ATF3	1.210089	9.71E-08	2.49E-05
SERPING1	1.465796	1.22E-07	2.85E-05
IFITM3	1.196708	1.70E-07	3.62E-05
ISG15	1.584807	1.71E-07	3.62E-05
BATF2	1.329895	2.02E-07	3.88E-05
DNAPTP6	1.48339	3.19E-07	5.34E-05
IFI27	2.979257	3.37E-07	5.44E-05
IFIT1	1.477969	1.34E-06	0.000156
MMP28	−1.2842	4.41E-09	3.33E-06
RBPMS2	−1.08485	1.74E-08	7.65E-06
LOC644532	−1.07012	2.89E-08	1.23E-05
LOC650761	−1.22368	4.15E-08	1.46E-05
LOC389816	−1.1437	7.92E-08	2.16E-05
CD248	−1.46395	1.25E-07	2.85E-05
HS.567460	−1.68229	3.89E-07	6.19E-05
HS.449276	−1.00543	8.39E-07	0.00011
LRRN3	−1.12993	7.12E-05	0.002464

### Co-Expression Networks

**Figure 1:**
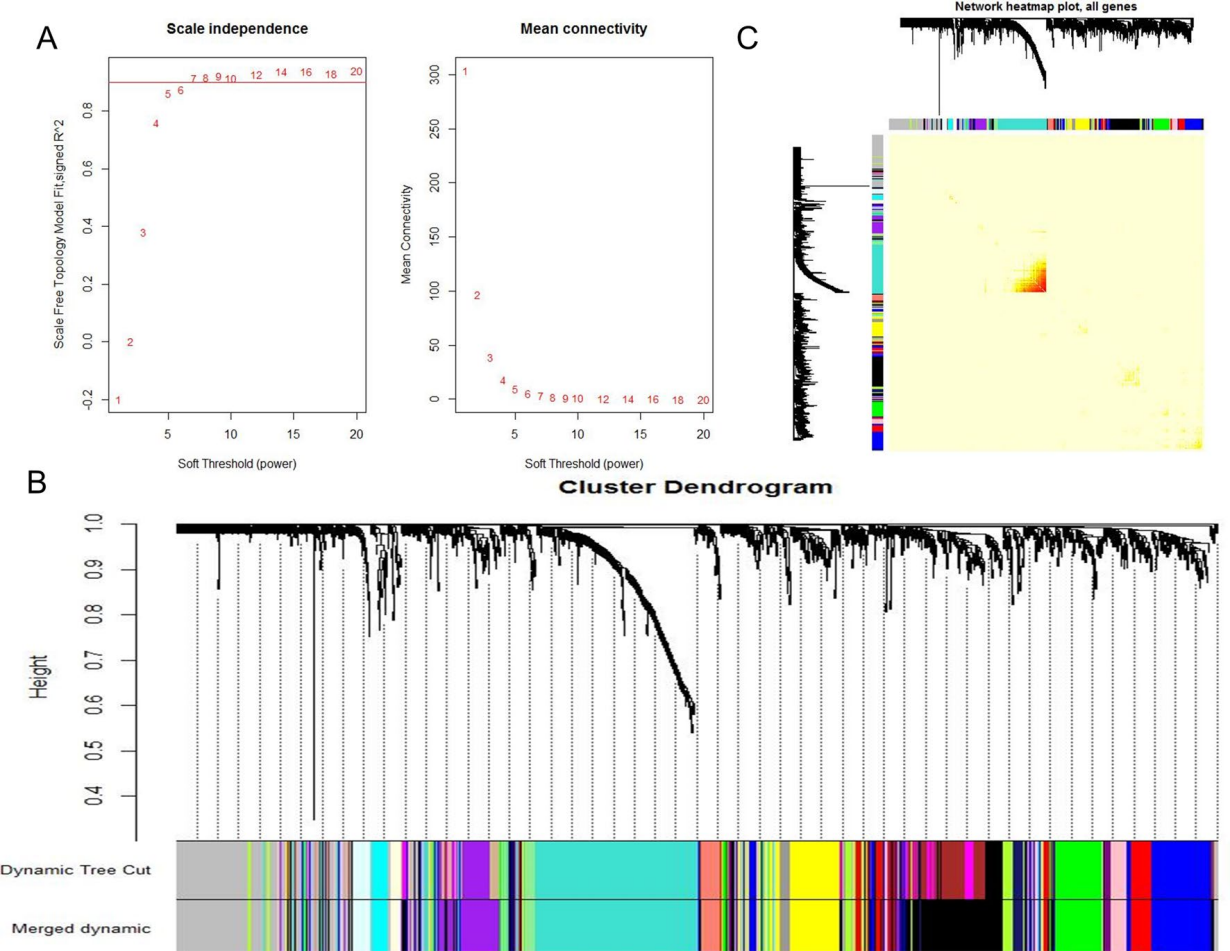
WGCNA revealed gene co-expression networks in the whole peripheral blood of 190 SS patients. **(A)** Analysis of the scale-free fit index for various soft-thresholding powers (Left) and analysis of the mean connectivity for various soft-thresholding powers (Right); **(B)** Clustering dendrogram of differentially expressed genes related to SS in the whole peripheral blood tissues of 192 SS patients; **(C)** Network heatmap plot in the co-expression modules (The progressively saturated red colors indicated higher overlap among the functional modules.).

When 0.9 was used as the correlation coefficient threshold, the soft-thresholding power was selected as eight (**Figure 1A**). Through WGCNA analysis, 17 co-expression modules were constructed (**Figure 1B**). The module comprising most genes was the turquoise one, followed by the black module, the blue module, and the yellow module (**Figure 1B**). Moreover, these modules were independent of other modules (**Figure 1C**).

### Module-Trait Correlations in Sjögren’s Syndrome and Identification of Hub Genes

**Figure 2:**
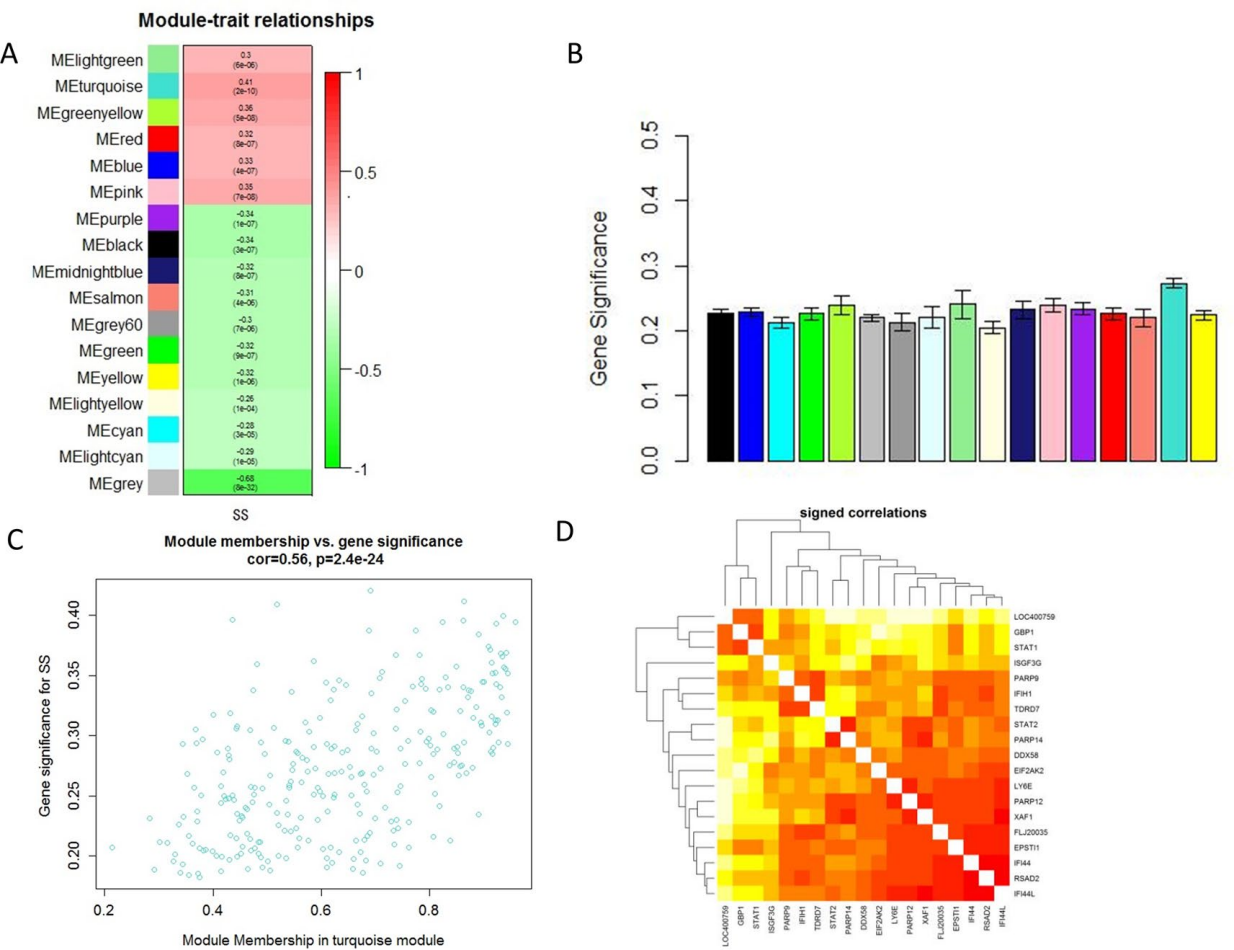
Main findings in the module-trait correlations analyses. **(A)** Heatmap between the correlation between modules and SS (Each cell contained the correlation coefficient and corresponding P value.); **(B)** Module significance values of those co-expression modules associated with SS (Module significance value indicated the summary of gene significance of all genes in each module, and different colors of column indicated different modules.); **(C)** The gene significance for SS in the turquoise module (One dot represents one gene in the turquoise module.); **(D)** Top 19 genes with high gene significance for SS were intensively correlated to each other.

Module-trait correlations analyses showed that multiple modules were related to SS (**Figure 2A**). **Figure 2B** showed the summary of significance of all genes in each module related to SS. It clearly indicated that the turquoise module was most significantly associated with SS (**Figure 2B**). **Figure 2C** showed the significance of these genes in the turquoise module for SS (**Figure 2C**). Notably, some genes in the turquoise module such as GBP1, PARP9, EPSTI1, LOC400759, STAT1, STAT2, IFIH1, EIF2AK2, TDRD7, IFI44, PARP12, FLJ20035, PARP14, ISGF3G, XAF1, RSAD2,LY6E, IFI44L, and DDX58 had high gene significance for SS (**Figures 2C, D**). Besides, these genes mentioned above were also closely related to each other (**Figure 2D**). Thus these genes could be considered as hub genes.

### Functional Annotation of the Key Co-Expression Module

**Figure 3:**
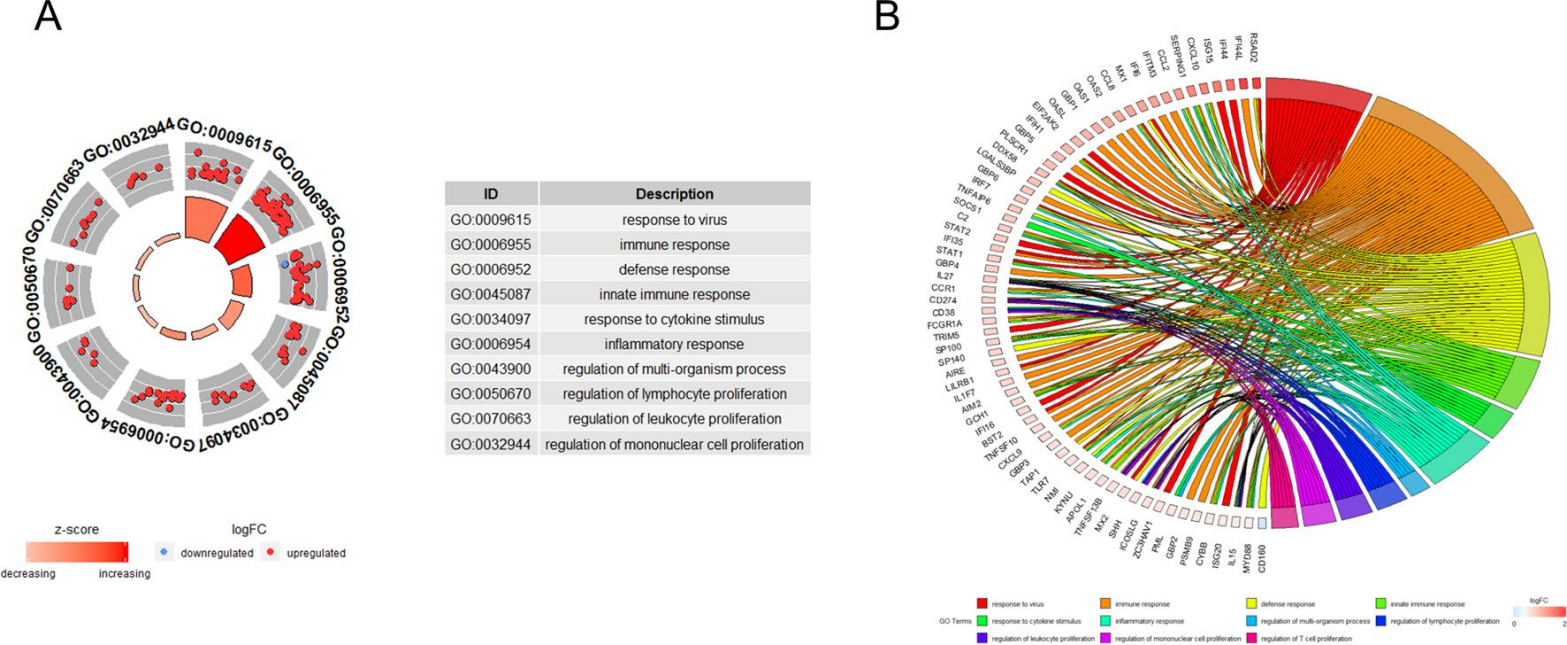
Functional analysis. **(A)** Gene ontology enrichment analysis of turquoise module genes. **(B)** Circos plot to indicate the relationship between genes and GO terms. Cross-examination of the relationship between these genes and GO biological process terms suggested that a substantial number of genes related to immune response were also enriched for other biological process such as defence response, response to cytokine stimulus and the inflammatory response.

GO functional enrichment analysis showed that the genes in turquoise module were mainly enriched in biological process being involved in response to virus, immune response, defense response, response to cytokine stimulus, and the inflammatory response (**Figure 3A**). Cross-examination of the relationship between these genes and GO biological process terms suggested that a substantial number of genes related to immune response were also enriched for other biological processes such as defense response, response to cytokine stimulus, and the inflammatory response, indicating that these genes could be related to multiple biological pathways orchestrating SS development (**Figure 3B**).

### Validation and Efficacy Evaluation of Hub Genes

**Figure 4:**
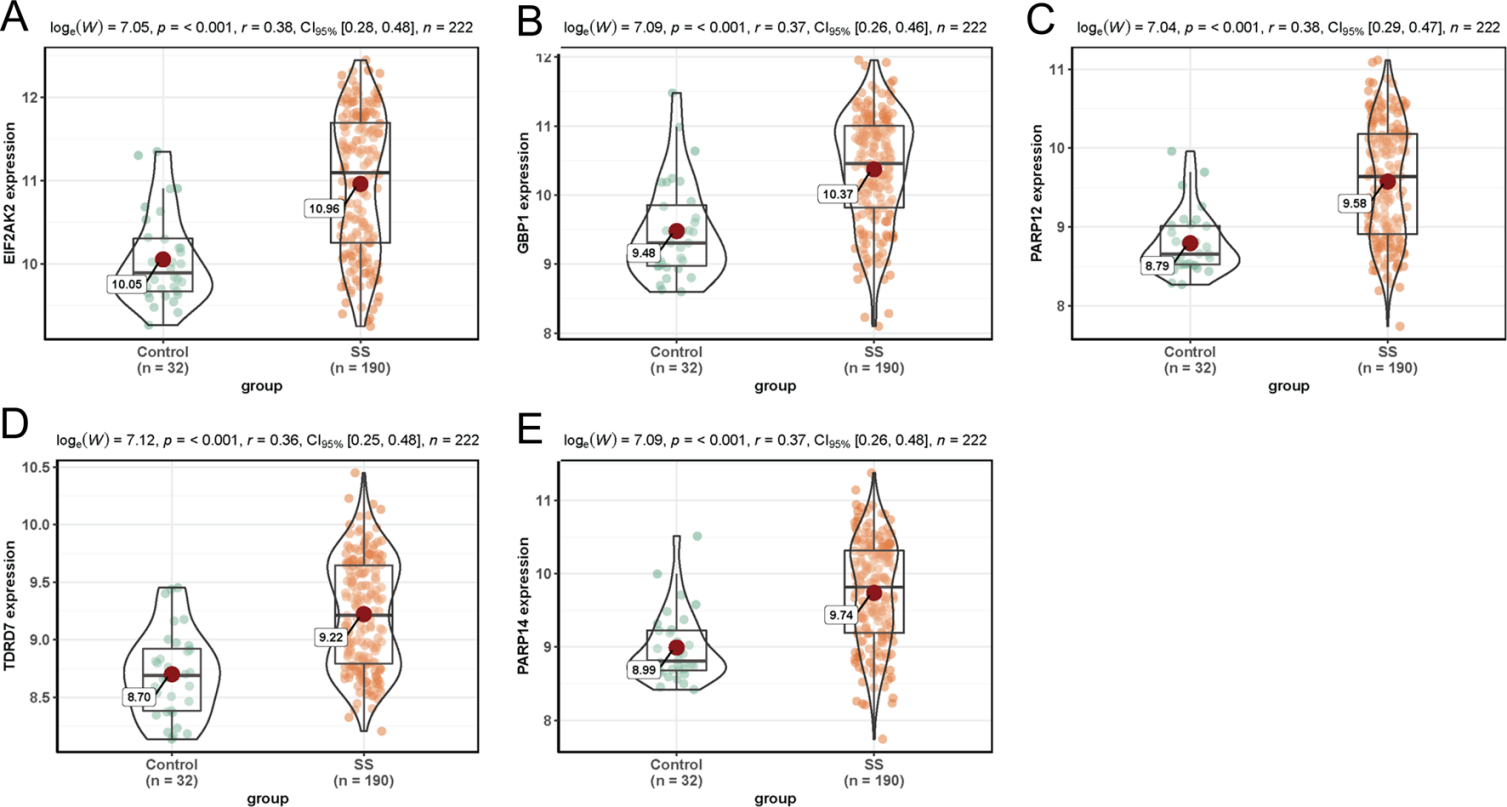
Expression of hub genes in dataset GSE51092. **(A–E)** Expression levels of EIF2AK2 **(A)**, GBP1 **(B)**, PARP12 **(C)**, TDRD7 **(D)**, and PARP14 **(E)** were significantly increased in SS patients.

**Figure 5:**
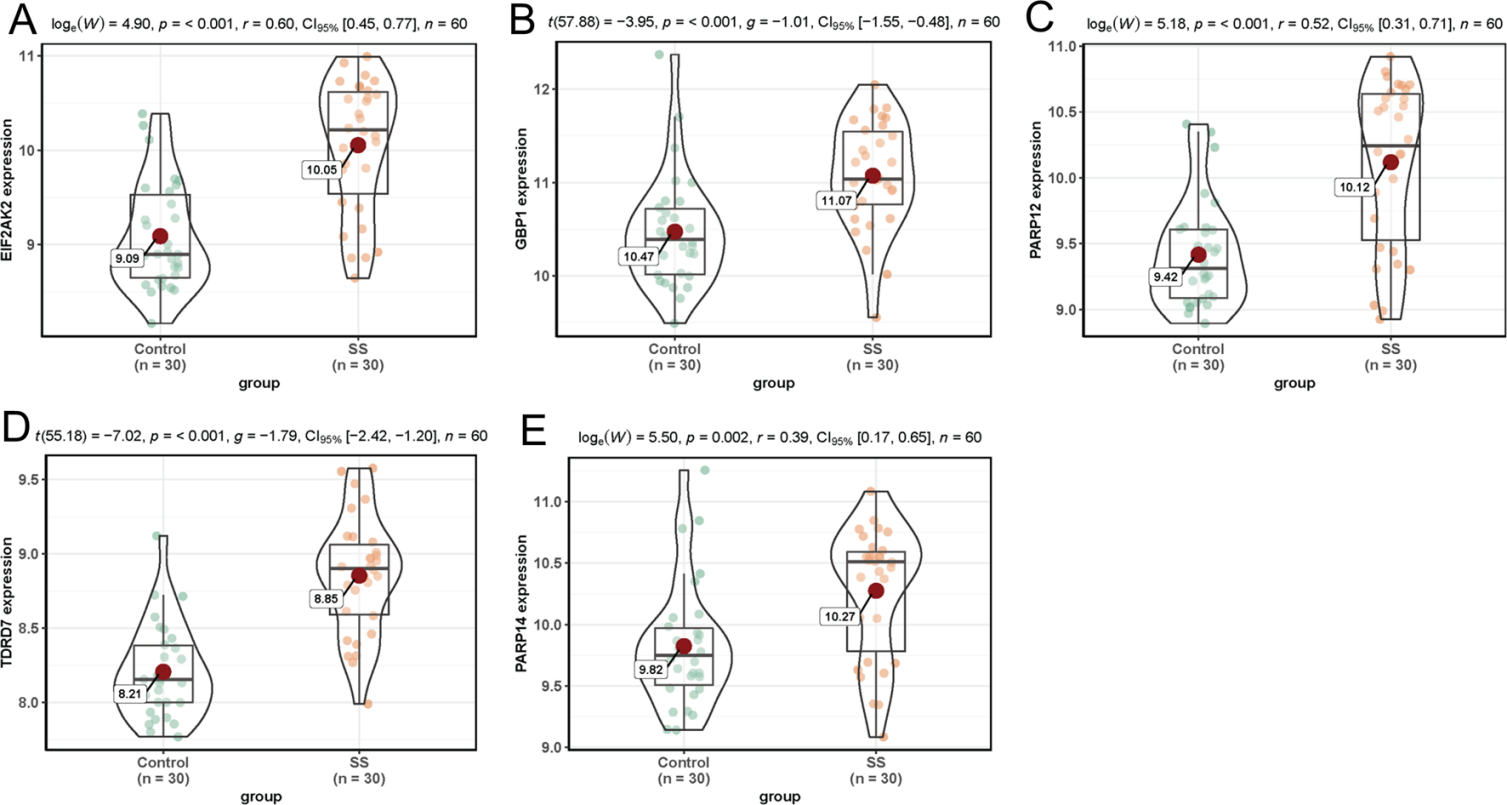
Validation of hub genes in dataset GSE84844. **(A**–**E)** Expression levels of EIF2AK2 **(A)**, GBP1 **(B)**, PARP12 **(C)**, TDRD7 **(D)**, and PARP14 **(E)** were significantly upregulated in SS patients.

**Figure 6:**
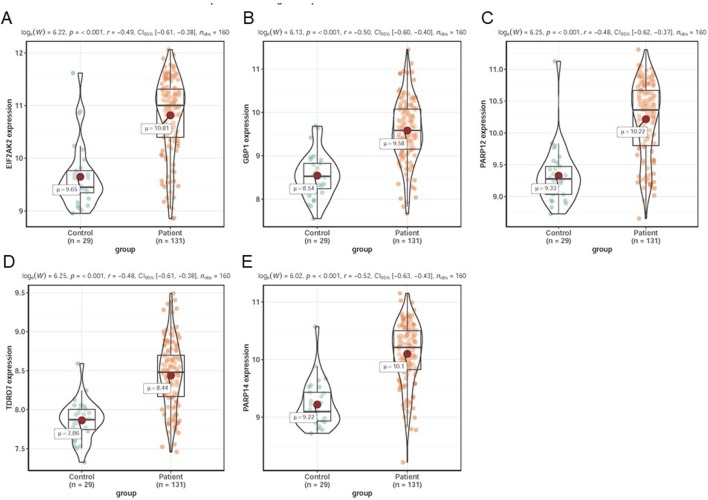
Validation of hub genes in dataset GSE66795. **(A–E)** Expression levels of EIF2AK2 **(A)**, GBP1 **(B)**, PARP12 **(C)**, TDRD7 **(D)**, and PARP14 **(E)** were significantly upregulated in SS patients.

**Figure 7:**
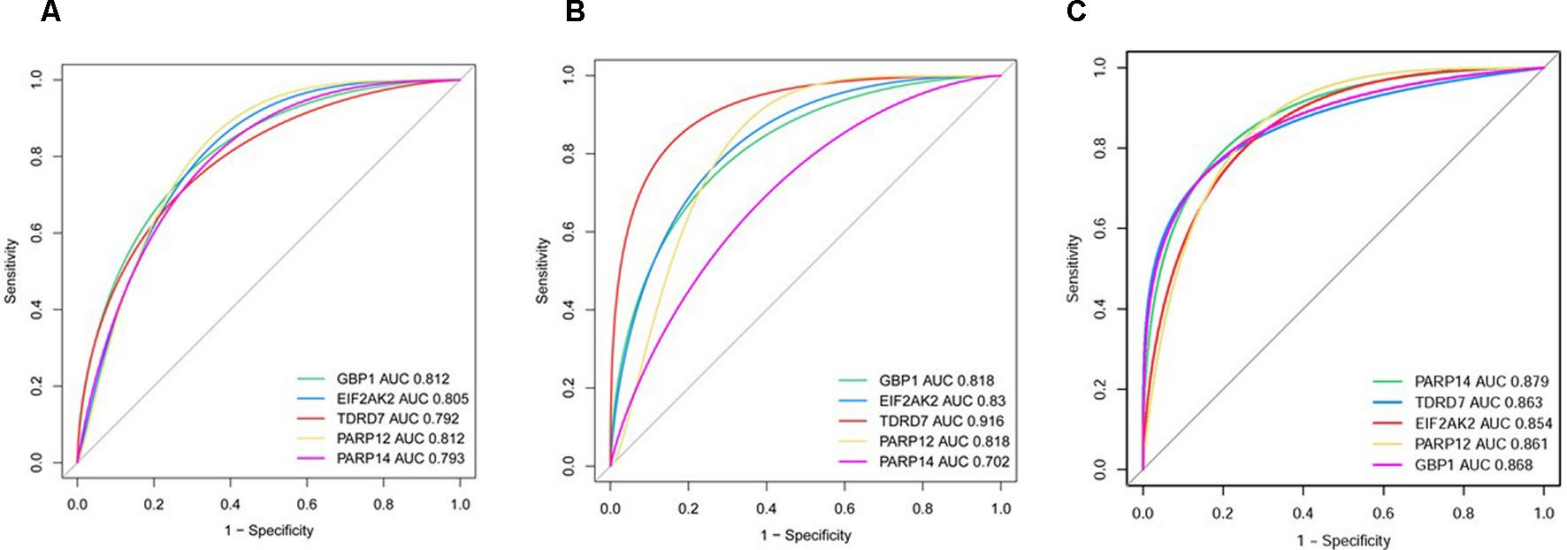
ROC curve of hub genes including EIF2AK2, GBP1, PARP12, PARP14, and TDRD7 in two datasets. **(A)** GSE51092 **(B)** GSE84844 **(C)** GSE66795.

In dataset GSE51902, the expression of five interested genes including EIF2AK2 (**Figure 4A**), GBP1 (**Figure 4B**), PARP12 (**Figure 4C**), TDRD7 (**Figure 4D**), and PARP14 (**Figure 4E**) was significantly increased in the SS patients. What’s more, the expression levels of the above five hub genes were investigated in another two datasets GSE84844 and GSE66795. As shown in **Figure 5**, the expression of EIF2AK2 (**Figure 5A**), GBP1 (**Figure 5B**), PARP12 (**Figure 5C**), TDRD7 (**Figure 5D**), and PARP14 (**Figure 5E**) was also significantly up-regulated (all P < 0.001) in the PBMCs of SS patients compared to controls in dataset GSE84844. **Figure 6** displayed that the expression trend of EIF2AK2 (**Figure 6A**), GBP1 (**Figure 6B**), PARP12 (**Figure 6C**), TDRD7 (**Figure 6D**), and PARP14 (**Figure 6E**) in dataset GSE66795 was the same as GSE84844. In addition, ROC curve was plotted and the area under the curve (AUC) was calculated to distinguish SS from controls, and every AUC of the five real hub genes was greater than 0.7 in datasets GSE51092 (**Figure 7A**), GSE 84844 (**Figure 7B**), and GSE66795 (**Figure 6C**).

### Gene Set Enrichment Analysis

**Figure 8:**
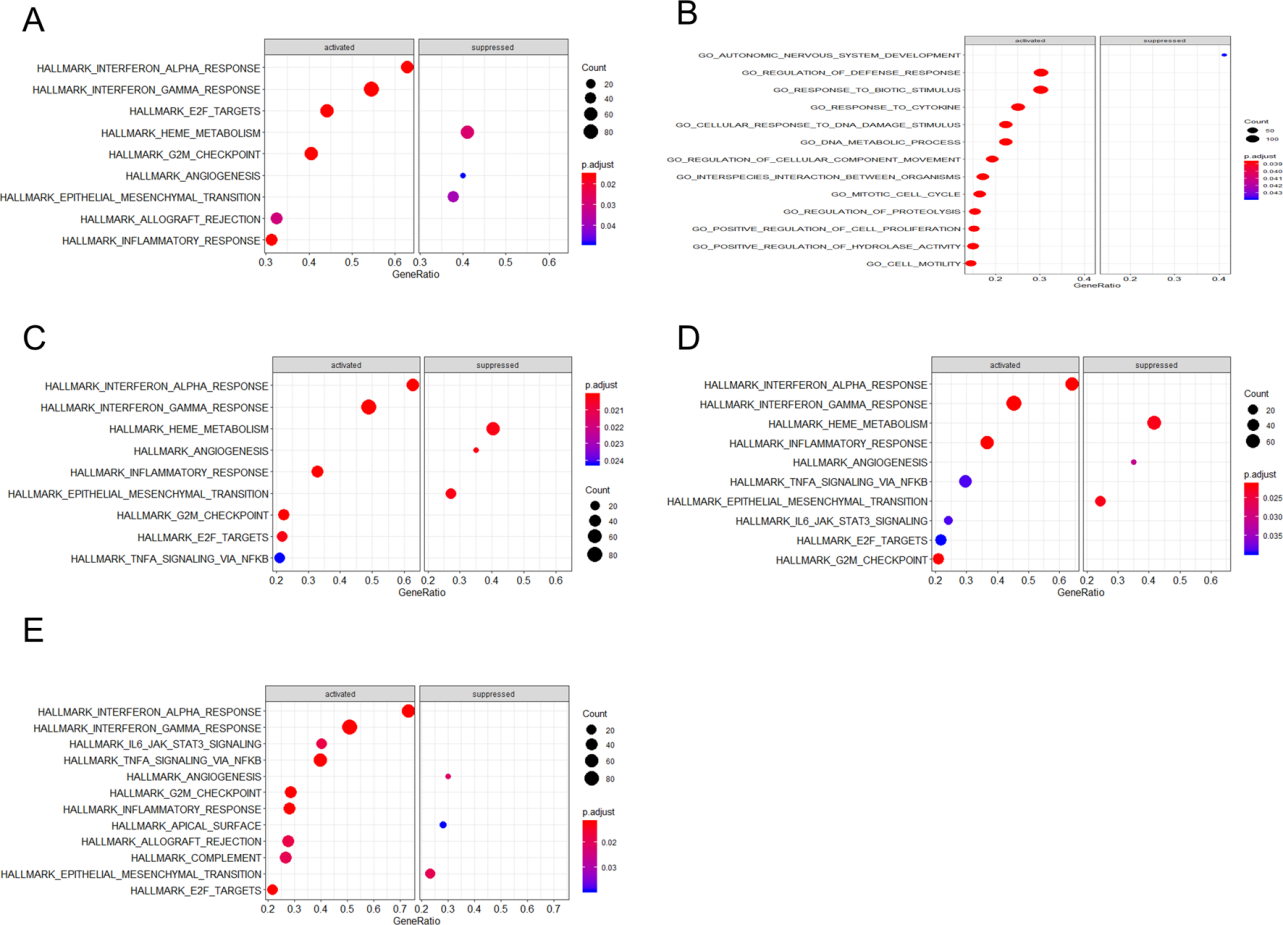
Gene set enrichment analysis (GSEA). The full list of gene sets enriched in samples with EIF2AK2 **(A)**, GBP1 **(B)**, PARP12 **(C)**, PARP14 **(D)**, or TDRD7 **(E)** highly expressed.

**Figure 9:**
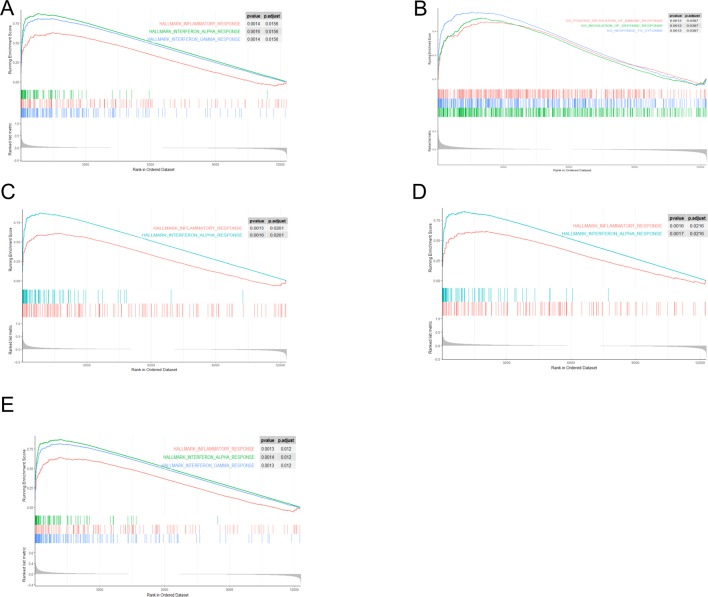
Gene sets related to immunity. Gene sets related to immunity enriched in samples with EIF2AK2 **(A)**, GBP1 **(B)**, PARP12 **(C)**, PARP14 **(D)**, or TDRD7 **(E)** highly expressed.

Through gene set enrichment analysis, we found the full list of gene sets enriched in samples with EIF2AK2 (**Figure 8A**), GBP1 (**Figure 8B**), PARP12 (**Figure 8C**), PARP14 (**Figure 8D**), or TDRD7 (**Figure 8E**) highly expressed. Then we selected the gene sets related to immunity among the full list to perform further analysis. Three gene sets were enriched in samples with highly expressed EIF2AK2 and TDRD7, including “inflammatory response,” “interferon α response,” and “interferon γ response”(**Figures 9A, E**). Similarly, in the samples with GBP1 highly expressed, “Regulation of the immune response,” “Regulation of the defense response,” and “response to cytokine” were enriched (**Figure 9B**). Moreover, gene sets “inflammatory response” and “interferon α response” were enriched in the samples with either PARP12 or PARP14 highly expressed (**Figures 9C, D**).

## Discussion

As far as we know, our study is the first one to apply WGCNA to build the SS-related gene-network with samples more than 200. Through WGCNA method, we constructed SS-related gene co-expression networks, and found several key gene co-expression modules and several hub genes related to the pathogenesis of SS. The results of this research present new insights into the molecular mechanism of SS development.

In the present study, a total of 17 co-expression modules were obtained by WGCNA analysis. Among them, the turquoise module was the main one involved in SS, containing 278 genes. Besides turquoise, there were several other co-expression modules in SS, such as black, blue, and yellow modules. Therefore, the etiology of SS involves complex genetic networks.

GO functional enrichment analysis is very powerful and widely used to classify biological entities into functional related groups (Rue-Albrecht et al., 2016). In this study, we also applied GO analysis to elucidate the biological functions of genes in the turquoise module. The results showed that the turquoise module was mainly enriched in response to virus, immune response, defense response, response to cytokine stimulus, and the inflammatory response. These findings further confirmed the involvement of multiple immune processes and cytokines in the pathogenesis of SS.

Among the 17 modules, the turquoise module is the key one involved in SS pathogenesis. Besides, among the 278 genes in the turquoise module, some genes had greater significance for SS than others, such as GBP1, PARP9, EPSTI1, LOC400759, STAT1, STAT2, IFIH1, EIF2AK2, TDRD7, IFI44, PARP12, FLJ20035, PARP14, ISGF3G, XAF1, RSAD2, LY6E, IFI44L, and DDX58. These genes could be regarded as hub genes. In addition, they can play important roles in certain co-expression module. However, their mechanisms in SS are largely unclear.

Most of these hub genes are type I interferons inducible genes. To date, the molecular mechanism of SS is not well clarified, but cumulative evidence showed that SS patients have an activated interferon type I response (Bave et al., 2005; Vakaloglou and Mavragani, 2011). It was demonstrated by enhanced interferon type I activity and increase expression of interferons-regulated genes in SS patients (Emamian et al., 2009). IFN, EPSTI1, STAT1, and IFI44L were identified in a meta-analysis among the top 20 differentially expressed genes associated with SS (Song et al., 2014). The interferon type I signature is present in over half of the SS patients and related to disease activity and the presence of autoantibodies (Kirou et al., 2005; Brkic et al., 2013). One study has found an upregulation of IFIH1, RSAD2, and DDX58 in plasmacytoid dendritic cells and monocytes of Interferon-positive SS patients and a downregulation of IFIH1 and DDX58 in Interferon-negative SS patients (Maria et al., 2017). Interferon type I inducible genes IFI44, IFI44L, LY6E, and XAF1 were all increased in patients with SS (Brkic et al., 2013). PARP9, also, an IFN-induced gene, was found with distinct hypomethylation and upregulation in CD19^+^ B cells of SS patients. Our study further confirmed that interferon type I signature was involved in the pathogenesis of SS. However, seven (GBP1, LOC400759, EIF2AK2, TDRD7, PARP12, FLJ20035, and PARP14) of these genes have not been studied in SS. Among them, in dataset GSE51902 (Lessard et al., 2013), the expression of EIF2AK2, GBP1, PARP12, PARP14, and TDRD7 was significantly increased in the SS patients. What’s more, the expression levels of the above five hub genes were validated in another dataset. We found that their expression was also significantly up-regulated in the PBMCs of SS patients compared to controls in dataset GSE84844 (Tasaki et al., 2017). Although these two studies used different platforms for gene expression analysis and were conducted on very distinct populations, the expression trends of these five genes were not affected. The reason may be that the data were normalized or standardized. It also suggests that the expression of these five genes is universal in different ethnic groups. Of course, their expression and related function also need to be elucidated in more different races in the future.

In addition, our study revealed that immune response, inflammatory response, response to cytokine stimulus, and regulation of lymphocyte proliferation were involved in the pathogenesis of SS based on the functional analysis. In fact, a study has found that TH2 helper cells cytokines dominate in the early lesions of SS, while TH1 helper cells cytokines are related to later stages of the disorder (Mitsias et al., 2002). Studies have demonstrated an association of IL-17, IL-18, IL-22, IL-36α, and IL-37 with the pathophysiology of SS (Ciccia et al., 2015;Xin et al., 2015; Liuqing et al., 2017;Matsui and Sano, 2017). Also, CD4^+^ T lymphocytes comprise majority of the glandular infiltration in SS (Nair and Singh, 2017). However, the exact roles of these cytokines and lymphocyte in SS are not clear.

Although the present study is the first to investigate the coexpression gene networks associated with SS using WGCNA analysis with large sample size, our study also has limitations. On one hand, we did not further study the exact mechanism of the identified hub genes in SS. On the other hand, in our WGCNA analysis and validation of hub genes, we used the data from three different studies. These three studies used different platforms for gene expression analysis and were conducted on very distinct populations. Thus, the expression of these hub genes still needs to be investigated in more different races.

In summary, our study finds involvement of the key gene co-expression module, hub genes and some functional biological pathways related to immune response, inflammatory response and cytokines in the pathogenesis of SS. These findings provide new insights into the development of SS, although the exact molecular mechanism of hub genes and functional pathway in SS still need to be further explored.

## Data Availability Statement

Publicly available datasets were analyzed in this study. This data can be found here: https://www.ncbi.nlm.nih.gov/gds/?term=GSE51092.

## Author Contributions

J-aZ designed the study. QY, ZS, BW, and QQ performed data analysis. QY wrote the manuscript. J-aZ revised the manuscript. All the authors read and approved the final manuscript.

## Funding

The present work was supported by grants from the National Natural Science Foundation of China (No. 81873636).

## Conflict of Interest

The authors declare that the research was conducted in the absence of any commercial or financial relationships that could be construed as a potential conflict of interest.
